# Body composition parameters for predicting the efficacy of neoadjuvant chemotherapy with immunotherapy for gastric cancer

**DOI:** 10.3389/fimmu.2022.1061044

**Published:** 2022-12-08

**Authors:** Guang-Tan Lin, Jiao-Bao Huang, Ju-Li Lin, Jian-Xian Lin, Jian-Wei Xie, Jia-Bin Wang, Jun Lu, Chao-Hui Zheng, Chang-Ming Huang, Ping Li

**Affiliations:** ^1^ Department of Gastric Surgery, Fujian Medical University Union Hospital, Fuzhou, China; ^2^ Key Laboratory of Ministry of Education of Gastrointestinal Cancer, Fujian Medical University, Fuzhou, China; ^3^ Fujian Key Laboratory of Tumor Microbiology, Fujian Medical University, Fuzhou, China

**Keywords:** gastric cancer, neoadjuvant immunotherapy, body composition, tumor regression grade (TRG), adverse events

## Abstract

**Background:**

Immune checkpoint inhibitors are increasingly used in neoadjuvant therapy for locally advanced gastric cancer. However, the effect of body composition on the efficacy of neoadjuvant therapy has not been reported.

**Methods:**

The computed tomography (CT) images and clinicopathological data of 101 patients with locally advanced gastric cancer who received neoadjuvant chemotherapy combined with immunotherapy (NCI) from 2019 to 2021 were collected. The CT image of L3 vertebral body section was selected, and the body composition before and after the neoadjuvant treatment was calculated using the SliceOmatic software, mainly including skeletal muscle index (SMI), subcutaneous adipose index (SAI), and visceral adipose index (VAI). The relationship between body composition and the efficacy and adverse events of NCI was analyzed.

**Results:**

Of the 101 patients, 81 with evaluable data were included in the analysis. Of the included patients, 77.8% were male; the median age of all the patients was 62 years, and the median neoadjuvant therapy cycle was three. After the neoadjuvant therapy, 62.9% of the tumors were in remission (residual tumor cells ≤ 50%), and 37.1% of the tumors had no remission (residual tumor cells>50%). Moreover, 61.7% of the patients had treatment-related adverse events (TRAEs), and 18.5% had immune-related adverse events (irAEs). After neoadjuvant therapy, the body mass index (from 23 to 22.6 cm^2^/m^2^, p=0.042), SAI (from 34.7 to 32.9 cm^2^/m^2^, p=0.01) and VAI (from 32.4 to 26.8 cm^2^/m^2^, p=0.005) were significantly lower than those before treatment, while the SMI had no significant change (44.7 vs 42.5 cm^2^/m^2^, p=0.278). The multivariate logistics regression analysis revealed that low SMI (odds ratio [OR]: 3.23,95% confidence interval [CI]: 1.06–9.81, p=0.047), SMI attenuation (△SMI) ≥ 1.8(OR: 1.45,95%CI: 1.20–3.48, p=0.048), and clinical node positivity (OR: 6.99,95%CI: 2.35–20.82, p=0.001) were independent risk factors for non-remission. Additionally, high SAI is an independent risk factor for irAEs (OR: 14, 95%CI: 1.73–112.7, p=0.013).

**Conclusion:**

Low SMI and △SMI≥1.8 are independent risk factors for poor tumor regression in patients with advanced gastric cancer receiving NCI. Patients with a high SAI are more likely to develop irAEs.

## Introduction

Gastric cancer remains one of the major malignant tumors causing cancer-related deaths, and its mortality ranks fourth among all malignant tumors worldwide (1).Even with surgery or adjuvant radiotherapy and chemotherapy, the 5-year survival rate of patients with stage II gastric cancer is 61%–63%, while that of patients with stage III decreased to 30%–35% (2). immune checkpoint blockers (ICB) therapy has made great progress in the treatment of patients with advanced gastric cancer. Preclinical studies and some phase II clinical studies have provided theoretical support and clinical evidence for neoadjuvant chemotherapy combined with immunotherapy (NCI) for locally advanced gastric cancer (3). The CheckMate-649 study has revealed that compared with chemotherapy alone, navulizumab combined with chemotherapy significantly improved overall survival (OS) and progression-free survival (PFS) in patients with metastatic gastric cancer and gastroesophageal junction (GEJ) cancer, and was recommended as the first-line treatment for subgroups with PD-L1 combined positive score ≥ 5 (4). Meanwhile, the NEONIPIGA study has demonstrated that nivolumab and ipilimumab-based neoadjuvant therapy is feasible and associated with no unexpected toxicity and a high pathologic complete response (PCR) rate in patients with mismatch repair deficient/microsatellite instability resectable GEJ adenocarcinoma (5). Previous studies in our center have confirmed that NCI has a higher gastric resection rate and better tumor regression than chemotherapy alone for locally advanced gastric cancer (6).

Weight loss and body composition change are common symptoms of patients with malignant tumors, and they are often related to poor prognosis (7), especially in gastric cancer (8). Lee et al. have reported that postoperative muscle attenuation and surgery-induced low skeletal muscle index(L-SMI) are prognostic factors for survival in patients with GC (9). Park et al. have reported that the decrease of muscle and subcutaneous adipose and visceral adipose was significantly related to the decrease of RFS and OS (10). Several recent studies have discovered no evident change in body composition of patients with locally advanced gastric cancer during neoadjuvant chemotherapy, although they have reported that L-SMI and muscle attenuation are related to the effect and postoperative complications of neoadjuvant chemotherapy (11, 12). However, whether this phenomenon exists in patients with NCI remains unknown, and the effects on body composition after immunotherapy have not been reported. Therefore, this study aimed to evaluate the changes of body composition and its effects on tumor remission and immune-related adverse events (irAEs) in patients with gastric cancer receiving NCI.

## Methods

### Study population and data collection

This study retrospectively analyzed the data of 101 patients with locally advanced gastric cancer who received NCI in the Department of Gastric Surgery, Fujian Medical University Union Hospital from January 2019 to April 2021. The inclusion criteria were as follows: age 18–75 years; with primary gastric adenocarcinoma confirmed by histopathology, clinical stage: cT2–4, lymph node N0~N3, and no distant metastasis (M0); received no chemotherapy (radiotherapy) or other antineoplastic therapy within 6 months; with computed tomography (CT) scans available during diagnosis and before operation; and without evidence of distant metastasis, such as liver metastasis or peritoneal implantation metastasis after laparoscopic exploratory surgery. Meanwhile, the exclusion criteria were as follows: with cancer complicated with malignant diseases of other organs; with evidence of peritoneal dissemination or distant metastasis (including intraoperative exploration after neoadjuvant therapy); and with history of gastrectomy or endoscopic submucosal dissection. In total, 11 cases without operation, six cases with incomplete CT data, and three cases with abdominal implant metastasis were excluded. Finally, 81 patients were included in the analysis (**Figure S1**). The study was reviewed and approved by the Ethics Committee of Fujian Medical University Union Hospital.

### Body composition

Body components comprise adipose and non-adipose tissues, the former including subcutaneous adipose, visceral adipose, and intermuscular adipose tissues, and the latter including the muscles, bones, and internal organs (13). A single CT image of the third lumbar vertebra (L3) was selected to quantify muscle and adipose features, as the anatomical location was closely related to body volume (13). According to the standard Hounsfield unit (HU) range, skeletal muscle cross-sectional area (SMT, −29–150 HU), visceral adipose tissue (VAT, −15–50 HU), and subcutaneous adipose tissue (SAT, −190–30 HU) were quantified. A researcher (L.J.X.) tackled how to accurately capture the image in the middle of L3 and segment muscle and adipose tissue. All CT images without any patient information were then analyzed using the SliceOmatic version 5.0 (TomoVision) (14). The measured value of each body component (square centimeter) divided by the square meter of height was converted into an index (SMI, visceral adipose index [VAI], and subcutaneous adipose index [SAI]) (15, 16),. Body mass index (BMI) was calculated by dividing the weight by height squared. In the analysis, the patients were divided into groups according to BMI as follows: low BMI (<25 kg/m^2^) group and high BMI (≥25 kg/m^2^) group. According to the results of Martin (17), the male patients with BMI < 25 kg/m^2^ and SMI < 43 cm/m^2^ (or BMI ≥ 25 kg/m^2^ and SMI < 53 cm/m^2^) were considered L-SMI, while the female patients with SMI < 41 cm/m^2^ were considered L-SMI regardless of BMI. According to the relationship between the SAI and incidence of irAEs, the patients were divided into two groups as follows: high subcutaneous adipose group (H-SAI) and low subcutaneous adipose group (L-SAI). Additionally, we used the median to classify the VAI because no threshold for VAI has been clinically established. The △SMI, △VAI, and △SAI represent the changes of SMI, VAI, and SAI before and after neoadjuvant therapy, respectively.

### Neoadjuvant therapy regimen

The neoadjuvant immunotherapy regimen is a fluorouracil-based chemotherapy combined with ICBs. The SOX/XELOX regimen generally comprised 2–4 cycles of SOX/XELOX regimen (18)(S-1 40–60 mg/m^2^ or capecitabine 1000 mg/m^2^, twice a day, days 1–14, and oxaliplatin 130 mg/m^2^ intravenous injection on the first day). The FOLFOX4 regimen comprised 2–4 cycles (19)(day 1: oxaliplatin 85 mg/m^2^, calcium folinate 200 mg intravenous drip for 2 h, fluorouracil 400 mg/m^2^ intravenous drip, 22 h intravenous drip of fluorouracil 600 mg/m^2^). ICBs were administered intravenously along with the chemotherapy cycle on the first day of chemotherapy (the drug dose was determined according to the patient’s body surface area, and the dose was reduced appropriately for patients with severe blood toxicity or non-blood toxicity). The next cycle of chemotherapy was repeated on the 22nd day. According to the criteria described by the Japan Gastric Cancer Association (JGCA), a whole abdominal CT scan was performed every 6–8 weeks to evaluate the response to neoadjuvant therapy, and improve the results of the relevant laboratory tests (including blood routine, liver and kidney functions, and tumor markers.) (20). The operation was performed at least 3 weeks after the completion of the neoadjuvant therapy. All surgical operations, including the extent of lymph node dissection, were performed in accordance with the guidelines of the JGCA (21), while staging was performed according to the tumor–node–metastasis classification (American Joint Committee on Cancer staging, 8th edition) (22). The Becker regression criteria were used to quantify the pathological reaction after treatment. The standard is based on the estimation of the percentage of living tumor cells relative to the tumor bed that can be recognized by the naked eye, and includes the following categories: TRG1a (no residual tumor cells), TRG1b (<10% residual tumor cells), TRG2 (10%–50% residual tumor cells), and TRG3 (>50% residual tumor cells) (23). In this study, TRG grade 1a/1b/2 was considered tumor remission (TR), and the TRG3 grade was considered non-tumor remission (non-TR).

### Adverse events

Treatment-related adverse events (TRAEs) were assessed according to the National Cancer Institute-General terminology Standard for adverse events (AEs) version 4.0 (24). TRAEs included events reported between the first administration and the last study 30 days after treatment. For further analysis, the toxicity was classified into grades I–II and III–IV. irAEs are defined as AEs associated with immunosuppressant exposure and in accordance with immune-related phenomena (25). TRAEs include general AEs and irAEs. Any delayed dose or early cessation of treatment recorded the result of significant toxicity (III–IV), which was defined as dose limited toxicity in this study.

### Nutritional support

As in previous studies, all the patients received nutritional risk screening using the Nutritional Risk Screening 2002 (NRS 2002), as recommended by the European Society for Clinical Nutrition and Metabolism (26), and developed personalized nutritional support therapy. Patients with an NRS score ≥ 3 were routinely provided oral nutritional supplements. For patients who were unable to meet their energy needs through oral feeding, enteral tube feeding and/or parenteral nutrition were provided (27). Additionally, all patients received a nutritional assessment every 2 weeks to adjust their nutritional support treatment until 1 week preoperatively.

### Statistical analysis

**Figure 1:**
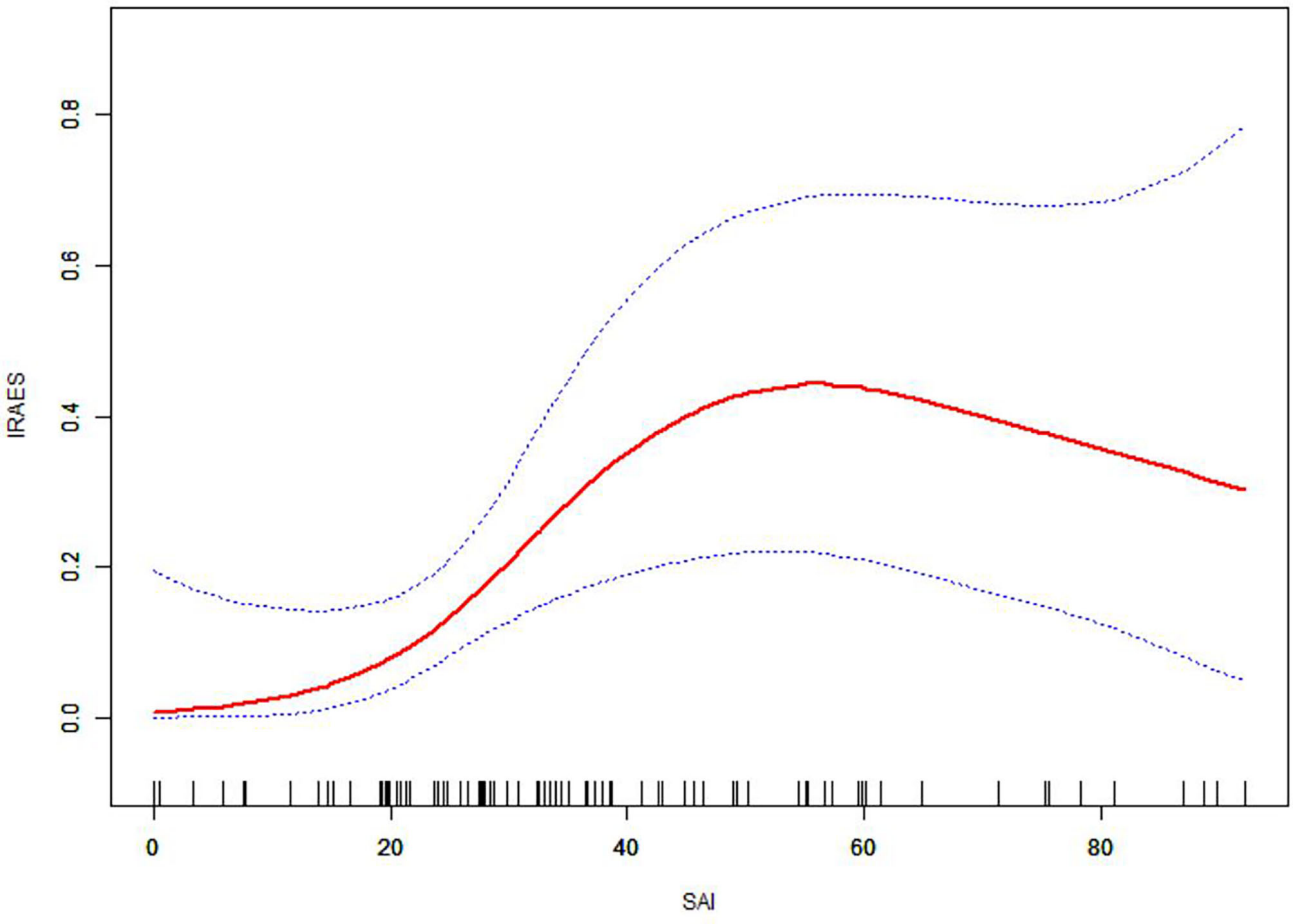
The relationship between subcutaneous adipose index and irAEs.

The main endpoint was pathological reaction. The secondary endpoints included TRAEs and irAEs. Normally distributed variables are described as the absolute number and percentage, mean, and standard deviation, and nonparametric variables are described as median and interquartile range. The classified variables were analyzed by double X^2^ test or Fisher’s exact test, the continuous variables were compared by Student’s t test, and the paired t-test was used before and after the comparison. The correlation among the parameters was analyzed by pearson correlation coefficient, and the cutoff point of △SMI was intercepted according to the maximum area under the receiver operating characteristic curve. According to the smooth curve, the relationship between SAI and irAEs is explored, and the potential confounding factors are adjusted (**Figure 1**). The experimental method was used to determine the relationship between the incidence of irAEs and SAI levels, move the test inflection point along predefined intervals, and detect the inflection point of the maximum model possibility. We further apply applied a two-stage linear regression model to test the threshold effect of SAI on irAEs according to the smoothing curve (**Table S2**). A logistic regression model was used for the univariate and multivariate analyses. Significance was set at p<0.05. All statistical analyses were conducted using the SPSS software version 22.0 and Empower Stats 2.0.

## Results

### General clinicopathological data of patients

Altogether, 81 patients who received NCI and underwent gastrectomy in the Fujian Medical University Union Hospital from January 2019 to April 2021 were included. Among them, 63 patients were male (77.8%), and 18 were female (22.2%). The median age of the patients was 62 years (57–67). The median preoperative neoadjuvant therapy cycle was three (3–4). Total gastrectomy and distal subtotal gastrectomy were performed in 64 (79%) and 17 (21%) cases, respectively. Postoperative complications occurred in 16 cases (19.7%), including pulmonary infection in 10 cases (12.3%), abdominal infection in four cases (4.9%), and anastomotic leakage in two cases (2.5%). Postoperative pathological stages included PCR in 12 cases (14.8%), ypI stage in 11 cases (13.6%), ypII stage in 20 cases (24.7%), and ypIII stage in 38 cases (46.9%). According to the Lauren classification, the intestinal type was recorded in 68 cases (84%) and the diffuse and mixed type in 13 cases (16%). After neoadjuvant therapy, 51 cases (62.9%) had TR, and 30 cases (37.1%) had non-TR. According to the set threshold, the body components were divided into the L-SMI group with 56 cases (69.1%), high SMI (H-SMI) group with 25 cases (30.8%), L-SAI group with 47 cases (57.1%), H-SAI group with 34 cases (41.9%), low VAI (L-VAI) group with 40 cases (49.4%), and high VAI (H-VAI) group with 41 cases (50.6%) (**Table 1**).

**Table 1:** General characteristics of patients.

Characteristics		Total n=81
Gender, No. (%)	Male	63 (77.8)
	Female	18 (22.2)
Age, y , (IQR)		62 (57-67)
ECOG, No. (%)	0	45 (55.6)
	≥1	36 (44.5)
Body mass index, No. (%	Low	58 (71.6)
	High	23 (28.4)
Days from diagnosis to surgery,mean (IQR)		124 (38-165)
Neoadjuvant therapy cycle,median (IQR)		3 (3-4)
Lauren classification, No. (%)	Intestinal	68 (84)
	Diffuse/Mixed	13 (16)
Type of gastrectomy, No. (%)	Total	64 (79)
	Distal	17 (21)
ypTNM stage, No. (%)	pCR	12 (14.8)
	I	11 (13.6)
	II	20 (24.7)
	III	38 (46.9)
Tumor regression grade, No. (%)	TR	51 (62.9)
	non-TR	30 (37.1)
R category, No. (%)	R0	76 (93.8)
	R1	5 (6.2)
Postoperative complication, No. (%)	Yes	16 (19.7)
	No	65 (80.3)
Treatment-related adverse events, No. (%)	Yes	50 (61.7)
	No	31 (38.3)
Immune-related adverse events, No. (%)	Yes	15 (18.6)
	No	66 (81.4)
Skeletal muscle index, (cm2/m2)	Low	56 (69.1)
	High	25 (30.9)
Subcutaneous adipose index, (cm2/m2)	Low	47 (58.1)
	High	34 (41.9)
Visceral adipose index, (cm2/m2 )	Low	40 (49.4)
	High	41 (50.6)

IQR, Interquartile range; ECOG, Eastern cooperative oncology group.

### Changes of body composition during neoadjuvant therapy

**Figure 2:**
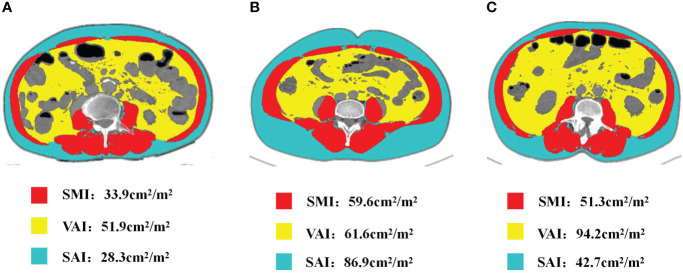
The portal phase computed tomography image of the L3 level was used to measure the body composition. Red: SM, Skeletal muscle;Yellow:VAT, Visceral adipose tissue;Blue:SAT, Subcutaneous adipose tissue; **(A)** L-SMI, Low skeletal muscle index; **(B)** H-SAI, High subcutaneous adipose index; **(C)** H-VAI,High visceral adipose index.

**Figure 3:**
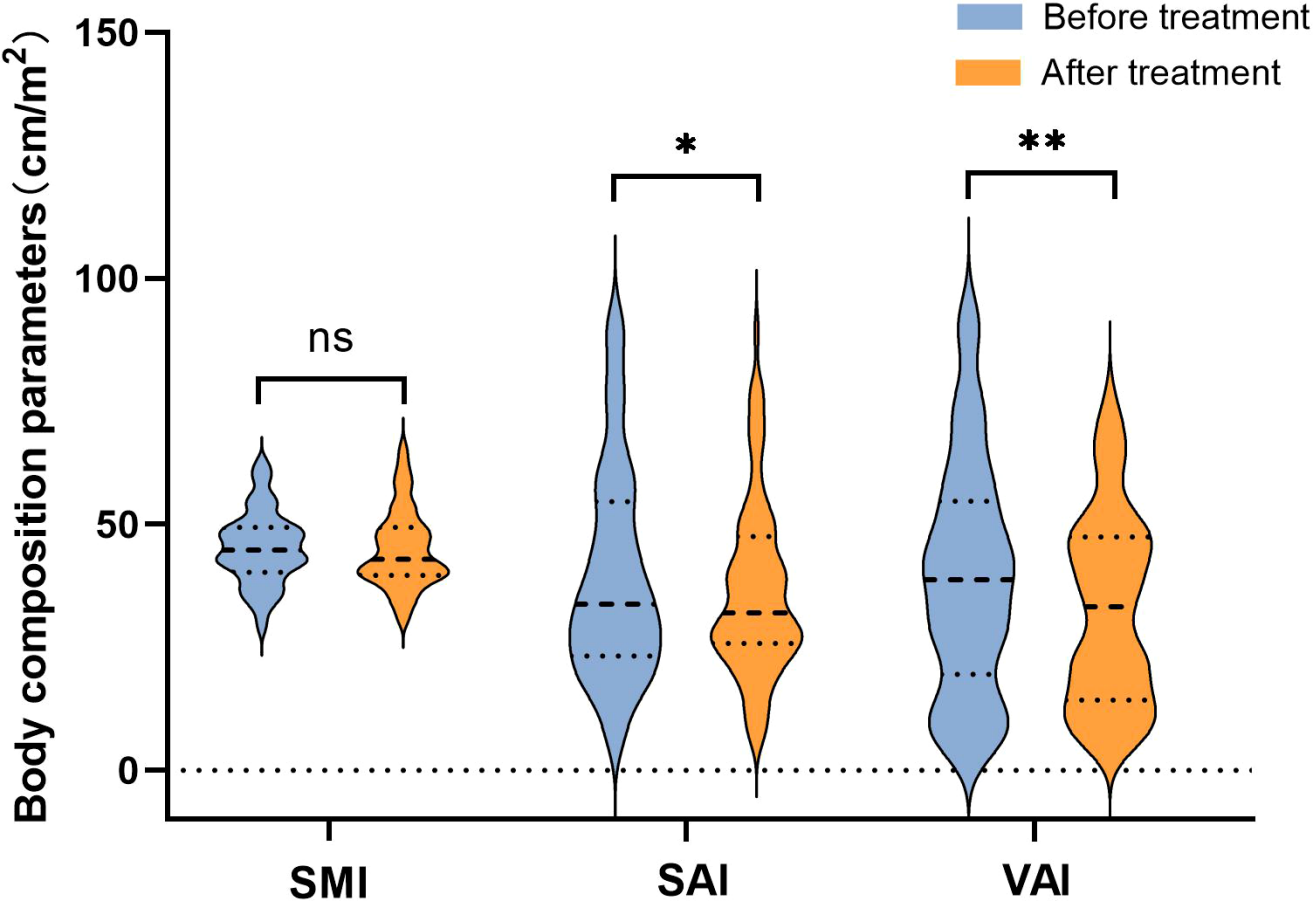
Changes of body composition parameters before and after neoadjuvant immunotherapy. “*”means p < 0.05; "**" means p < 0.01.

**Figure 2** presents a representative L3 plane CT image segmentation legend with the patient’s baseline state. Panel A depicts the representative segmentation of L-SMI, panel B indicates the representative segmentation of H-SAI, and panel C demonstrates the representative segmentation of H-VAI. After neoadjuvant therapy, the BMI (from 23 to 22.6 kg/m^2^, p=0.042), SAI (from 34.7 to 32.9 cm^2^/m^2^, p=0.01), and VAI (from 32.4 to 26.8 cm^2^/m^2^, p=0.005) were significantly lower than those before treatment, while the SMI had no significant change (44.7 vs 42.5 cm^2^/m^2^, p=0.278)(**Figure 3**). We also analyzed the relationship between the body components of patients at baseline and serum nutritional markers. The median of albumin (ALB) levels was 38g/L (33–41) before treatment and 37g/L (34–42) after treatment. The difference between the two groups before and after treatment was significant (p=0.047) (**Table 2**). Before neoadjuvant therapy, the SMI was positively correlated with ALB levels (Pearson’s=0.3, p=0.009), but not with SAI and VAI (SAI: Pearson’s=−0.151, p=0.626; VAI: Pearson’s=0.119, p=0.856).

**Table 2:** Changes of body composition and albumin in neoadjuvant therapy.

Outcomes	Median (IQR)	Pearson Correlation	P Value
SMI, cm2/m2		0.766	0.278
Pre	44.7 (40.1-49.6)		
Post	42.5 (39.5-49.2)		
ΔSMI	-0.3 (-2.8-2.2)		
SAI, cm2/m2		0.889	**0.010**
Pre	34.7 (21.5-55.1)		
Post	32.9 (23.6-47.7)		
ΔSAI	-0.6 (-9.1-2.6)		
VAI, cm2/m2		0.872	**0.005**
Pre	32.4 (12.0-52.1)		
Post	26.8 (13.2-46.9)		
ΔVAI	-0.5 (-13.6-3.2)		
BMI, kg/m2		0.888	**0.042**
Pre	23 (20.8-25.2)		
Post	22.6 (20-25.1)		
ΔBMI	-0.2 (-1.1-0.6)		
ALB, g/L			
Pre	38 (33-41)	0.696	**0.047**
Post	37 (34-42)		
△ALB	2 (-3-6)		

SMI, Skeletal muscle index; SAI, Subcutaneous adipose index; VAI, Visceral adipose index; BMI, Body mass index; ALB, albumin; △SMI, Post SMI—PreSMI, △ SAI, Post SAI—Pre SAI, △ VAI, Post VAI—Pre VAI, △ BMI, Post BMI—PreBMI, △ ALB, Post ALB—Pre ALB; IQR, Interquartile range. Bold means p < 0.05

### Relationship of pathological TR and body composition

Compared with the TR group, the patients in the non-TR group were younger (63 years vs 60 years, p=0.029) and had more patients in stage III (27.5% vs 80%, p=0.001). No significant differences in sex, interval between diagnosis and operation, neoadjuvant treatment cycle, surgical methods, postoperative complications, and AEs were observed between the two groups. In terms of body composition, the non-TR group had more patients with L-SMI (60.8% vs 83.3%, p=0.046), and no significant differences in SAI, VAI, and BMI were noted between the two groups (p>0.05). During the neoadjuvant therapy, the SMI of patients in the non-TR group decreased more (△SMI: 0.5 vs −1.5, p=0.05), although the SAI and VAI did not change significantly (p>0.05) (**Table 3**).

**Table 3:** Comparison between two groups of general data and body composition.

	TR (n=51)	non-TR (n=30)	P.value
Gender, No. (%)			0.854
Male	40 (78.4)	23 (76.7)	
Female	11 (23.6)	7 (23.3)	
Age, y , (IQR)	63 (57-69)	60 (56-57)	**0.029**
ECOG, No. (%)			0.877
0	28 (54.9)	17 (56.7)	
≥1	23 (45.1)	12 (43.3)	
Days from diagnosis to surgery, mean (IQR)	124 (45-151)	122 (39-163)	0.860
Neoadjuvant therapy cycle,median (IQR)	3 (3-4)	4 (3-4.5)	0.301
Lauren classification, No. (%)			0.133
Intestinal	46 (90.2)	22 (73.3)	
Diffuse/Mixed	5 (8.8)	8 (26.7)	
Type of gastrectomy, No. (%)			0.769
Total	41 (80.4)	23 (76.7)	
Distal	10 (19.6)	7 (23.3)	
ypTNM stage, No. (%)			**0.001**
pCR	12 (23.5)	0 (0)	
I	10 (19.6)	1 (3.3)	
II	15 (29.4)	5 (16.7)	
III	14 (27.5)	24 (80)	
R category, No. (%)			0.891
R0	48 (94.1)	28 (93.3)	
R1	3 (5.9)	2 (6.7)	
Postoperative complication, No. (%)			0.554
Yes	12 (23.5)	4 (13.3)	
No	39 (76.5)	26 (86.7)	
TRAEs, No. (%)			0.353
Yes	30 (58.8)	20 (66.7)	
No	21 (41.2)	10 (33.3)	
irAEs, No. (%)			0.555
Yes	8 (15.7)	7 (23.3)	
No	43 (84.3)	23 (76.7)	
**Body composition parameters**			
SMI,No. (%)			**0.046**
Low	31 (60.8)	25 (83.3)	
High	20 (39.2)	5 (16.7)	
SAI,No. (%)			0.516
Low	21 (41.2)	13 (43.3)	
High	30 (58.8)	17 (56.7)	
VAI,No. (%)			0.961
Low	22 (43.1)	15 (50)	
High	29 (56.9)	15 (50)	
BMI,No. (%)			0.411
Low	35 (68.6)	23 (76.7)	
High	16 (31.4)	7 (23.3)	
△SMI (IQR)	0.5 (-2.6-2.5)	-1.5 (-3.3-0.8)	**0.05**
△SAI (IQR)	-1.2 (-7.7-4.8)	-3.9 (-9.5-1.4)	0.189
△VAI (IQR)	-4.6 (-15.2-3.0)	-4.3 (-8.5-3.9)	0.907
△BMI (IQR)	-0.1 (-1.1-0.8)	-0.3 (-1.2-0.3)	0.218

ECOG, Eastern cooperative oncology group; SMI, Skeletal muscle index.

SAI, Subcutaneous adipose index; VAI, Visceral adipose index; BMI, Body mass index; IQR, Interquartile range; irAEs, Immune-related adverse events; TRAEs, Treatment-related adverse events. Bold means p < 0.05

### SMI and its changes predict TR

**Table 4** summarizes the results of the univariate and multivariate logistic analyses of body composition and their changes in patients with tumor regression before neoadjuvant therapy. Among them, the L-SMI (odds ratio [OR]: 3.45, 95% confidence interval [CI]: 2.06–6.81, p=0.041), and SMI ≥ 1.8 (OR: 1.38, 95%CI: 1.09–2.89, p=0.037) were risk factors for non-TR. After adjusting for age, Eastern Cooperative Oncology Group score, cT, cN and other clinical-related factors, the multivariate logistic analysis revealed that L-SMI (OR: 3.23, 95%CI: 1.06–9.81, p=0.047), SMI ≥ 1.8 (OR: 1.45, 95%CI: 1.20–3.48, p=0.048), and clinical node positivity (cN+) (OR: 6.99, 95%CI: 2.35–20.82, p=0.001) were independent risk factors for non-TR.

**Table 4:** Univariate and multivariable analysis of the relationship between body composition with non-TR.

	Univariable		Multivariable analysis	
	OR(95%CI)	p	OR(95%CI)	p
Age	0.95(0.90-1.00)	0.053	0.91(0.89-1.01)	0.058
Gender				
male				
female	0.89(0.61-1.73)	0.812		
ECOG				
0				
≥1	0.97(0.37-2.58)	0.906		
Lauren classification				
Intestinal				
Diffuse/Mixed	4.56(0.88-5.89)	0.736		
Pretreatment cT stage				
T2	Ref.			
T3				
T4	1.56(0.56-4.39)	0.431		
Pretreatment cN stage				
N0				
N+	6.74(2.33-19.44)	**0.001**	6.99(2.35-20.82)	**0.001**
Tumor location				
Upper	Ref.			
Middle				
Lower				
Mixed	0.45(0.31-6.82)	0.345		
Tumor differentiation				
Low/Middle				
High	0.88(0.67-5.44)	0.494		
SMI(Low vs High)	3.45(2.06-6.81)	**0.041**	3.23(1.06-9.81)	**0.047**
SAI(Low vs High)	1.47(0.44-4.9)	0.529		
VAI(Low vs High)	0.99(0.96-1.04)	0.955		
BMI(Low vs High)	0.43(0.07-2.74)	0.369		
ΔSMI(<1.8vs≥1.8)	1.38(1.09-2.89)	**0.037**	1.45(1.20-3.48)	**0.048**
ΔSAI	1.01(0.98-1.05)	0.101		
ΔVAI	0.84(0.75-1.66)	0.457		
ΔBMI	0.94(0.58-1.51)	0.792		

ECOG, Eastern cooperative oncology group; SMI, Skeletal muscle index; SAI, Subcutaneous adipose index; VAI, Visceral adipose index; BMI, Body mass index. Bold means p < 0.05.

### SAI is a predictor of AEs related to immunotherapy

In this study, 50 cases (61.7%) of TRAEs were recorded, of which 32 cases (39%) were grade III–IV TRAEs. Meanwhile, 15 cases (18.5%) had irAEs, of which seven cases (8.9%) were grade III–IV irAEs, including three cases of abnormal liver function, one case of interstitial pneumonia, two cases of maculopapular rash, and one case of immune colitis,irAEs are recorded separately (**Table S1**). No grade V AEs and drug withdrawal due to AEs were recorded.

Logistics analysis found that SMI(OR: 3.54,95%CI: 0.66-6.45,p=0.891), SAI (OR: 2.24,95%CI: 0.79-2.53, p= 0.119), VAI (OR: 0.56,95%CI: 0.46-1.64, p= 0.215) had no significant correlation with TRAEs (**Table S2**). However, when analyzing the relationship among SMI, SAI, VAI and irAEs, we found a nonlinear relationship between the incidence of SAI and irAEs.(**Figure 1**). As SAI reaches a turning point (28.5 cm^2^/m^2^), the risk of irAEs increases (**Table S3**). The incidence of irAEs in the H-SAI group (SAI ≥ 28.5 cm^2^/m^2^) was 29.8%. The incidence of irAEs in the L-SAI (SAI < 28.5 cm^2^/m^2^) group was 3%. **Table 5** presents the results of the univariate and multivariate logistics analyses of body composition and its changes before neoadjuvant therapy. The H-SAI, H-BMI, and cN+ were related to the occurrence of irAEs. Further multivariate analysis revealed H-SAI as an independent risk factor for irAEs (OR: 14, 95%CI: 1.73–112.7; p=0.013). Among them, the incidence of abnormal liver function increased mainly (aspartate aminotransferase increased at 29.6% vs 8.9%, p=0.043; alanine aminotransferase increased at 25.9% vs 2.8%, p=0.009) (**Figure S2**).

**Table 5:** Univariate and multivariable analysis of the relationship between body composition with irAEs.

irAEs	Univariable		Multivariableanalysis	
Character	OR(95%CI)	p	OR(95%CI)	p
Age	1.02 (0.94-1.09)	0.762		
Gender				
male				
female	0.59 (0.11-2.73)	0.472		
ECOG				
0				
≥1	0.92 (0.57-1.85)	0.885		
Pretreatment cT stage				
T2	Ref			
T3				
T4	3.6 (0.78-16.9)	0.101		
Pretreatment cN stage				
N0				
N+	2.30 (0.87-3.24)	0.096	1.79 (0.71-2.35)	0.137
Neoadjuvant therapy cycle	1.38 (0.91-2.09)	0.131		
SMI (Low vs High)	2.11 (0.37-4.57)	0.251		
SAI (Low vs High)	9.40 (0.99-88.2)	**0.050**	14 (1.73-112.7)	**0.013**
VAI (Low vs High)	0.89 (0.75-1.03)	0.129		
BMI (Low vs High)	3.95 (0.80-4.52)	0.085	2.84 (0.22-3.15)	0.199
ΔSMI (<1.8vs≥1.8)	0.92 (0.74-1.15)	0.458		
ΔSAI	1.05 (0.90-1.10)	0.180		
ΔVAI	0.84 (0.66-1.53)	0.213		
ΔBMI	0.39 (0.19-1.86)	0.190		

ECOG, Eastern cooperative oncology group; SMI, Skeletal muscle index; SAI, Subcutaneous adipose index; VAI, Visceral adipose index; BMI, Body mass index; irAEs, Immune-related adverse events.

## Discussion

Body composition is closely related to immunotherapy. Previous studies have reported on the relationship of body composition with the efficacy and toxicity of immunotherapy in melanoma (28, 29). However, the interaction between body composition and immunotherapy in gastric cancer remains unknown. In this study, VAI and SAI have been observed to decrease in varying degrees during NCI, which was different from the results previously reported for neoadjuvant chemotherapy alone (11, 12),. A follow-up analysis has revealed that L-SMI and SMI attenuation were independent risk factors for poor TR, and the H-SAI index was significantly correlated with irAEs.

The adverse effect of L-SMI on prognosis has been confirmed in various malignant tumors, which may be irrelevant to the mode of treatment. For example, Kudou et al. found that the survival rate of patients with low SMI after radical gastrectomy was significantly lower than that of normal SMI (30), while Kim et al. have reported that patients with L-SMI in immunotreated gastric cancer had shorter PFS (median, 1.4 months vs. 2.6 months; p = 0.026) (31).Similarity, the efficacy in patients with L-SMI was worse in this study. Sato et al. have suggested that cachexia is a manifestation of the high malignant potential of cancer, and that L-SMI is one of the characteristics of cachexia, thus making it related to poor chemotherapy response (32). However, the research of Chu et al.provides an interesting explanation in immunotherapy (29). Chu et al. believe that immunosuppressants, such as protein, are highly charged molecules. The antibody itself is extremely hydrophilic as most of the water in the human body is stored in the muscles (33). Thus, patients with the same weight but less muscle content may have lower utilization of antibodies, which eventually leads to poor efficacy.

Deshpande et al.elaborated how diet, inflammation and intestinal microbes play a role in determining the outcome of ICBs treatment (34).Malnutrition and inflammation may be the main drivers of low SMI (35, 36). Malnutrition usually leads to impaired immune response and is vulnerable to infection. Proper energy and balanced nutrition are essential for the establishment of a healthy immune system (37, 38).Inflammation is the main factor mediating skeletal muscle decomposition in cancer patients with low SMI (39, 40). Ali et al. proposed that L-SMI status induces upregulation of pro-inflammatory cytokines, including tumor necrosis factor and interleukin-1 and interleukin-6. These mediators may interfere with the immune system and tumor microenvironment, leading to adverse clinical outcomes (41).A number of studies have shown that intestinal microbiota profoundly affect the immunotherapy response of a series of malignant tumors (42). For example, in non-small cell lung cancer and renal cell carcinoma, fecal samples of patients receiving anti-PD-1 immunotherapy are rich in bacterial species (43). Therefore, malnutrition, changes in inflammatory state and disorders of intestinal microbial system in patients with low SMI may be the reasons for poor tumor regress after neoadjuvant immunotherapy.

Our results also demonstrate that SMI attenuation predicts worse efficacy. This has also been confirmed in other studies. Rutten et al. have identified that skeletal muscle loss in patients with ovarian cancer during neoadjuvant chemotherapy was significantly shorter than that in patients with unchanged OS (44). In our study, SMI attenuation was an independent factor for poor tumor response, although the cause of skeletal muscle attenuation during NCI has no exact mechanism. One possible explanation is that the strong malignant potential of the tumor leads to loss of appetite and systemic consumption during treatment. Accordingly, a high tumor malignant potential may itself be insensitive to neoadjuvant therapy.

Obesity is associated with the development of irAEs (45), which may be caused by chronic systemic inflammation caused by macrophages in adipose tissue (46). In fact, some studies have linked higher BMI to an increased risk of irAEs after immunotherapy (47).Bouchlaka et al. have reported that providing systemic irritant immunotherapy was well tolerated by mice with low fat content, although it eventually led to multiple organ pathological events and rapid death in rats with obesity (48). These results suggest that the toxic reaction is induced by a strong immune stimulation and is related to the pre-existing inflammatory environment of the patient. In this study, patients with H-SAI had a higher incidence of irAEs (29.8% vs 3.0%). Previous studies have demonstrated that the expression of leptin was positively correlated with the expression of PD-1 (49), and that the secretion of leptin in subcutaneous adipose was much higher than that in visceral adipose (50). Hence, patients with high subcutaneous adipose have higher expression of PD-1 and stronger immune stimulation, thus eventually leading to higher irAEs.

This study had some limitations. First, this was a single-center retrospective small sample study, which has unavoidable selective bias and can only represent the results from eastern countries. Nevertheless, this study is the largest report within the range of NCI for gastric cancer. Second, the treatment regimen in this study was chemotherapy combined with immunotherapy, not alone immunotherapy. At present, the best neoadjuvant therapy for locally advanced gastric cancer remains controversial. Therefore, our results can be a significant reference for patients using this regimen. Third, although immune-related adverse events were recorded separately in this study, they were not completely accurate in collection and differentiation because of the combination therapy.Finally, due to the short follow-up time, we only analyzed the short-term results of NCI. Next, we will continue to collect the long-term survival outcomes of these patients to verify the results of this study on tumor regression.

## Data Availability

The raw data supporting the conclusions of this article will be made available by the authors, without undue reservation.
