# Supplementary material for: Body composition parameters for predicting the efficacy of neoadjuvant chemotherapy with immunotherapy for gastric cancer
Source: Front Immunol. 2022 Dec 8;13:1061044. doi: 10.3389/fimmu.2022.1061044 (PMC9772614; doi:10.3389/fimmu.2022.1061044)
Supplement: Supplementary file 1 [file DataSheet_1.docx]

**Supplemental Content** eTables and eFigures

**Figure S1** Stduy flow

**Figure S2** Comparison of irAEs of different subcutaneous adipose index

**Table S1** Classification of TRAEs and irAEs in all patients

**Table S2** Univariate analysis of the relationship between body composition with treatment-related adverse events

**Table S3** Threshold effect analysis of irAEs on subcutaneous adipose index using piecewise linear regression


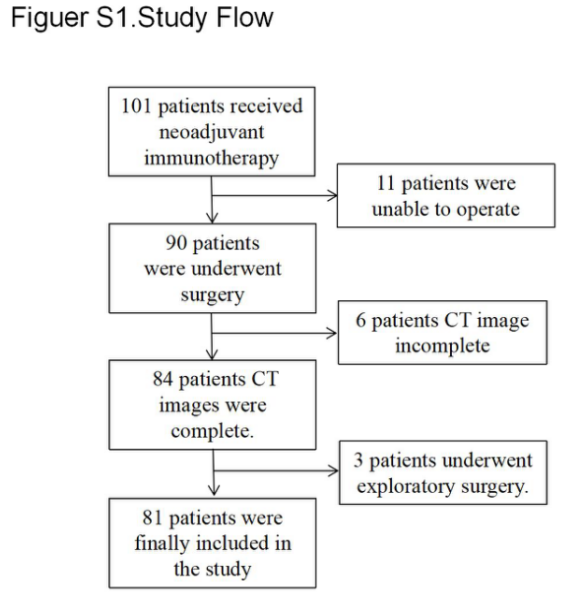


**Table S1** Classification of TRAEs and irAEs in all patients

| Adverse event | Any grade  n(%) | GradesI–II  n(%) | GradesIII–IVn(%) |
| --- | --- | --- | --- |
| Treatment-related adverse event | 50(61.7) | 36(44.4) | 32(39) |
| Any immune-related event | 15(18.5) | 13(16) | 7(8.6) |
| **General adverse event** | | | |
| Nausea | 33(40.7) | 30(37) | 3(3.7) |
| Diarrhoea | 25(30.9) | 21(25.9) | 4(4.9) |
| Peripheral neuropathy | 21(25.9) | 18(22.2) | 3(3.7) |
| Vomiting | 19(23.5) | 17(21) | 2(2.4) |
| Fatigue | 20(24.7) | 17(21) | 3(3.7) |
| Anaemia | 19(23.5) | 16(19.8) | 3(3.7) |
| Decreased appetite | 17(21) | 15(18.5) | 2(2.4) |
| Neutrophil count decreased | 18(22.2) | 13(16) | 5(6.2) |
| **Immune-related adverse event** | | | |
| Aspartate aminotransferase increased | 13(16) | 11(13.6) | 2(2.4) |
| Alanine aminotransferase increased | 11(13.6) | 10(12.3) | 1(1.2) |
| Interstitial pneumonia | 3(3.7) | 2(2.4) | 1(1.2) |
| Maculopapular rash | 6(7.4) | 4(4.9) | 2(2.4) |
| Colitis | 3(3.7) | 2(2.4) | 1(1.2) |
| Thyroid disorder | 1(1.2) | 1(1.2) | 0 |

**Table S2** Univariate analysis of the relationship between body composition with treatment-related adverse events

| **Treatment-related adverse events** | Univariable analysis | |
| --- | --- | --- |
| **Character** | OR(95%CI) | p |
| Age | 0.98（0.93-1.04） | 0.535 |
| Gender |  |  |
| male |  |  |
| female | 2.13（0.67-6.79） | 0.199 |
| ECOG |  |  |
| 0 |  |  |
| ≥1 | 0.85（0.30-2.43） | 0.763 |
| Pretreatment cT stage |  |  |
| T2 | Ref |  |
| T3 |  |  |
| T4 | 1.6（0.48-5.34） | 0.445 |
| Pretreatment cN stage |  |  |
| N0 |  |  |
| N+ | 0.94（0.29-3.03） | 0.916 |
| Neoadjuvant therapy cycle | 1.65(0.83-1.89) | 0.203 |
| SMI(Low vs High) | 3.54(0.66-6.45) | 0.891 |
| SAI(Low vs High) | 2.24(0.79-2.53) | 0.119 |
| VAI(Low vs High) | 0.56(0.46-1.64) | 0.215 |
| BMI(Low vs High) | 0.69(0.59-4.88) | 0.677 |
| ΔSMI(＜1.8vs≥1.8) | 0.96(0.81-1.13) | 0.738 |
| ΔSAI | 0.41(0.38-1.56) | 0.181 |
| ΔVAI | 1.07(0.22-1.12) | 0.177 |
| ΔBMI | 0.48(0.27-1.85) | 0.408 |
| **ECOG:** Eastern cooperative oncology group;**SMI**:Skeletal muscle index, **SAI:** Subcutaneous adipose index,**VAI:**Visceral adipose index,**BMI:**Body mass index； | | |

**Table S3** Threshold effect analysis of irAEs on subcutaneous adipose index using piecewise linear regression

| Inflection point of subcutaneous adipose index（cm2/m2） | Odds ratioa  (95 % CI) | P.value |
| --- | --- | --- |
| ＜28.5 | 0.87（0.56-3.15） | 0.654 |
| ≥28.5 | 2.34（1.89-60.36） | 0.011 |

**Figure S2** Comparison of irAEs of different subcutaneous adipose index


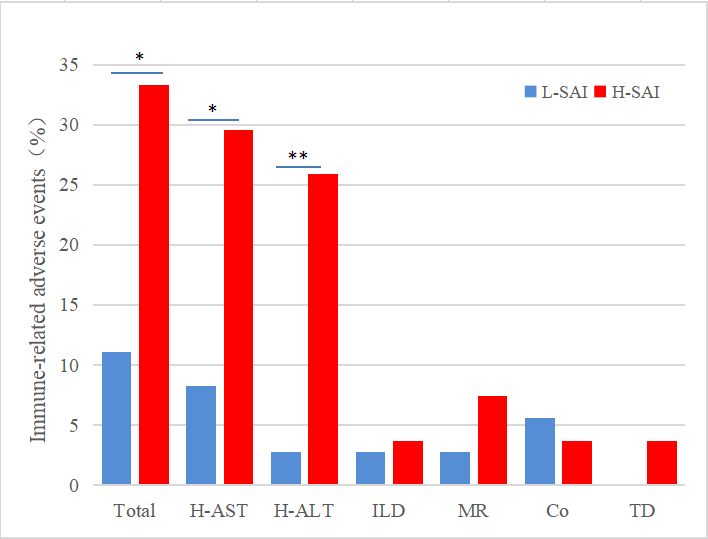


**H-AST:**Aspartate aminotransferase increased；

**H-ALT:** Alanine aminotransferase increased

**ILD**:Interstitial lung disease

**MR**:Maculopapular rash

**Co**:Colitis

**TD**:Thyroid disorder
